# Self-Motion Misperception Induced by Neck Muscle Fatigue

**DOI:** 10.3390/audiolres15050128

**Published:** 2025-10-02

**Authors:** Fabio Massimo Botti, Marco Guardabassi, Chiara Occhigrossi, Mario Faralli, Aldo Ferraresi, Francesco Draicchio, Vito Enrico Pettorossi

**Affiliations:** 1 Department of Medicine and Surgery, Human Physiology Section, Università Degli Studi di Perugia, Piazzale Gambuli 1, 06100 Perugia, Italy; fabio.botti@unipg.it (F.M.B.); mlookdowns@gmail.com (M.G.); chiara.occhi@outlook.it (C.O.); mario.far@hotmail.it (M.F.); aldo.ferraresi@unipg.it (A.F.); 2 Italian Society of Ergonomics and Human Factors, Via Ciro Menotti 11, 20129 Milano, Italy; fdraicch@gmail.com

**Keywords:** motion perception, muscle fatigue, neck muscle proprioception, balance, vestibular system

## Abstract

Background/Objectives: Previous research has demonstrated that the perception of self-motion, as signaled by cervical proprioception, is significantly altered during neck muscle fatigue, while no similar effects are observed when self-motion is signaled by the vestibular system. Given that in typical natural movements, both proprioceptive and vestibular signals are activated simultaneously, this study sought to investigate whether the misperception of motion persists during neck muscle fatigue when both proprioceptive and vestibular stimulation are present. Methods: The study evaluated the gain of the perceptual responses to symmetric yaw sinusoidal head rotations on a stationary trunk during visual target localization tasks across different rotational frequencies. In addition, the final localization error of the visual target was assessed following asymmetric sinusoidal head rotations with differing half-cycle velocities. Results: The findings indicated that even with combined proprioceptive and vestibular stimulation, self-motion perceptual responses under neck muscle fatigue showed a pronounced reduction in the gain at low-frequency stimuli and a notable increase in localization error following asymmetric rotations. Notably, spatial localization error was observed to persist after asymmetric stimulation conditioning in the light. Additionally, even moderate levels of muscle fatigue were found to result in increased self-motion misperception. Conclusions: This study suggests that neck muscle fatigue can disrupt spatial orientation, even when the vestibular system is activated, so that slow movements are inaccurately perceived. This highlights the potential risks associated with neck muscle fatigue in daily activities that demand precise spatial perception.

## 1. Introduction

Neck muscle fatigue occurs when the cervical muscles become overworked and exhausted, resulting in stiffness and a sensation of weakness or heaviness. Due to the crucial role of neck proprioception in regulating body position and motion detection [1,2,3,4,5,6,7], the effect of neck muscle fatigue may compromise, beyond the motor performance of the muscles, also balance [8], subjective vertical and horizontal perception [9], upper limb proprioception [10], posture [11,12], spatial orientation [13], and whole body pointing [14]. Recent studies [15,16] suggest that fatigue of the cervical muscles leads to altered perception of slow movements, resulting in inaccurate awareness of body position. This effect is suggested to arise from reduced proprioceptive signaling, as demonstrated in both animal [17,18] and human studies [19,20,21,22,23,24,25,26,27,28,29,30,31,32,33,34,35,36]. Specifically, ergoreceptive afferents are proposed to modulate proprioceptive input by inhibiting movement-related signals. This mechanism is further supported by findings that neck muscle vibration, which enhances neuromuscular spindle afferent discharge, can counteract the effects of fatigue [16].

Experimental evidence suggests that the erroneous self-motion perception induced by neck muscle fatigue may compromise balance and body positioning during physical activity, potentially increasing the risk of falls. However, this proprioceptive deficit can be mitigated when other sensory systems—such as the vestibular and visual systems—remain functional and can compensate.

Indeed, previous studies [15,16] have shown that the effects of neck muscle fatigue become apparent when self-motion perception is assessed through trunk rotation with a stationary head, which specifically engages neck proprioception. In contrast, when the entire body is rotated—stimulating only the vestibular system—no perceptual deficits have been observed, suggesting that vestibular-based self-motion perception remains unaffected by fatigue.

Since in everyday contexts, self-motion perception results from the integration of vestibular, visual, and proprioceptive cues, the proprioceptive deficit can be masked. Therefore, it may be important to examine how vestibular and visual signals could interfere with proprioceptive perceptual responses during fatigue. Furthermore, proprioceptive deficits have been observed only under conditions of intense fatigue, which may not reflect common real-world experiences. To better understand the practical relevance of fatigue-induced perceptual changes, we propose a study that incorporates vestibular stimulation through external perturbations, reduces fatigue intensity, and examines multisensory interactions.

To determine whether neck muscle fatigue affects motion perception in conditions involving both vestibular and proprioceptive input, we assessed self-motion perception during head rotations with a stationary trunk. Symmetric rotation trials were used to evaluate the gain of self-motion perception, while asymmetric rotations assessed self-positioning errors. These experiments were also conducted under illuminated conditions to test whether visual input mitigates the effects of fatigue. While most previous research has focused on fatigue induced by exhaustive effort, our study investigates whether even mild levels of fatigue can negatively influence motion perception, highlighting the broader implications of fatigue under less extreme conditions.

## 2. Method

### 2.1. Subjects

Ten right-handed participants (7 males, 3 females; age range: 19–60 years; mean age: 38.35 ± 12.45 years) were enrolled in the study. All participants had no history of neurological, visual, or vestibular disorders and provided informed consent prior to participation. Subjects varied in age, lifestyle, physical constitution, and athletic background. The experimental protocol adhered to the Declaration of Helsinki (1964) and was approved by the local ethics committee (University of Perugia, Perugia PG, Italy, protocol nr. 2018-06R).

### 2.2. Experimental Setting

#### 2.2.1. Neck Proprioceptive and Vestibular Sinusoidal Rotation

**Figure 1:**
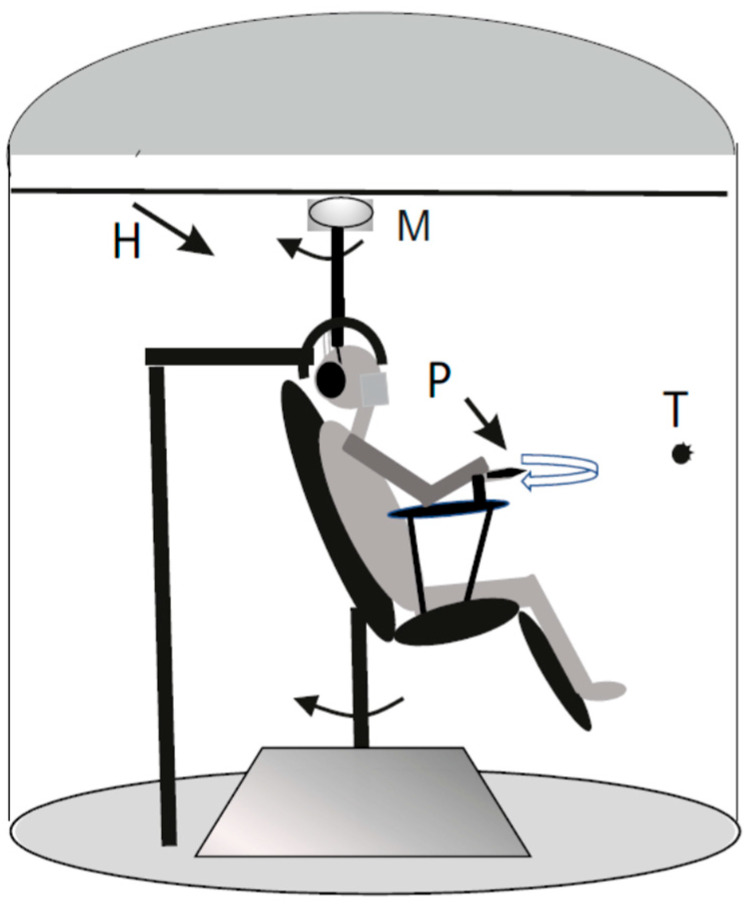
Depicts the experimental setup. Participants were rotated in darkness under symmetric or asymmetric sinusoidal conditions while tracking (arrow) a previously viewed visual target (T) using a pointer (P). The rotations (arrows) of the chair and of the head holder (H) by the DC motor (M) allowed selective stimulation of vestibular and/or neck proprioceptive systems. Trunk rotation was secured using restraining straps.

Participants were seated in a sound-attenuated cabin on a motorized rotating chair (horizontal plane) driven by a DC motor (Powertron, Contraves, Charlotte, NC, USA) and controlled via an angular velocity encoder (0.01–1 Hz, 1% accuracy) (Figure 1).

A head holder positioned the head 30° downward to align the horizontal semicircular canals with the plane of rotation [37]. We used three experimental conditions: Condition C (for stimulating neck proprioception only): The trunk was rotated while the head remained stationary. Condition V (for stimulating vestibular stimulation only): The entire body, including the head, was rotated. Condition C + V (for stimulating both vestibular + proprioception): The head was rotated while the trunk remained stationary. The head was moved by a ceiling-mounted motor connected to a helmet.

#### 2.2.2. Symmetric Rotation

Sinusoidal yaw stimuli (40° amplitude at 0.05, 0.1, 0.2, 0.5, 1 Hz) were administered. Symmetric stimuli assessed perceptual gain (perceptual amplitude/stimulus amplitude), while asymmetric stimuli assessed perceptual responses under contrasting half-cycle velocities [38]. Although stimulation primarily targeted vestibular and proprioceptive systems, slight activation of cutaneous receptors could not be ruled out.

#### 2.2.3. Asymmetric Rotation

The asymmetric stimulus consisted of two half-cycles with the same amplitude (40°) but differing in frequency: one fast half-cycle (Fast HC) at 0.39 Hz and one slow half-cycle (Slow HC) at 0.09 Hz. Peak acceleration was 120°/s^2^ and 7°/s^2^ for the fast and slow HCs, respectively, with corresponding peak velocities of 47°/s and 11°/s—both exceeding vestibular activation thresholds (~0.5–0.8°/s^2^ for VOR, 1.1–1.5°/s^2^ for perception) [39].

Asymmetric rotation was implemented by introducing an 80% asymmetry into a 0.15 Hz sinusoidal waveform. This was accomplished using custom software developed in Labview (National Instruments, Austin, TX, USA), which controlled the DC motor of the rotating chair. Head and trunk positions were recorded using an infrared video camera mounted on the cabin ceiling. The camera tracked two pairs of reflective infrared markers: one pair placed on the head (at the bregma and 3 cm posterior to it) and the other on the shoulders (at the left and right acromion).

#### 2.2.4. Self-Motion Perception Recording

Self-motion perception was assessed using a psychophysical tracking procedure [39,40,41,42]. Prior to rotation, subjects fixated on a visual target—a light spot with a 1 cm diameter—projected onto the cabin wall 1.5 m in front of them. The target was extinguished immediately before rotation onset and re-illuminated at the end of the stimulus. During the rotation, performed in complete darkness, participants were instructed to continue imagining and mentally tracking the fixed target with their eyes closed.

The tracking task was executed using a pointer mounted on a support fixed to the rotating platform, located 25 cm from the subject’s body axis and 100 cm above the platform surface. The pointer was connected to a precision potentiometer to measure movements.

We evaluated perceptual responses during both symmetric and asymmetric sinusoidal rotations. The symmetric stimulus was used to determine perceptual gain, calculated as the ratio between the amplitude of the pointer tracking and that of the chair rotation. In contrast, the asymmetric stimulus was used to assess adaptive responses to motion stimuli with velocity contrasts and their associated fatigue effects.

During asymmetric stimulation, we analyzed the final position error (FPE), defined as the discrepancy between the perceived and actual target location after four full cycles. This error arose from differential perception of fast and slow half-cycles: participants consistently perceived Fast HCs more vividly than Slow HCs [38]. Consequently, the perceived final target position was biased in the direction of the Slow HC. The magnitude of the error reflected the sensory system’s differential responsiveness to motion velocity, as well as the influence of adaptive mechanisms that enhance responses to faster stimuli while diminishing responses to slower ones. This adaptive mechanism is a result of the conditioning that every asymmetrical cycle induced in the subsequent perceptual responses.

#### 2.2.5. Neck Extensor Muscle Fatigue

Neck extensor muscle fatigue was induced through isometric muscle contraction. Participants were positioned prone on a table, with their heads protruding and extending against gravity. An additional load, equivalent to 40% of the maximal voluntary contraction (MVC) of the neck extensor muscles, was applied to the head. The MVC was determined by measuring the maximum extension force exerted against a strain gauge during head extension. Participants maintained head extension at this load until voluntary exhaustion, as indicated by their reports. The time to exhaustion generally ranged from 8 to 10 min. Following the fatigue protocol, participants performed neck extension stretches by flexing the head downward to mitigate potential thixotropic effects on the musculature and neuromuscular spindles [23].

Three minutes after the conclusion of the fatigue procedure, the individuals were tested to evaluate the FPE. In separate experiments, to examine the effect of a minor level of fatigue on FPE, tests were performed at 1, 3, and 6 min after the beginning of the fatigue protocol.

Fatigue was assessed by recording the EMG activity of the posterior neck extensor muscles and by calculating a fatigue index using the following formula:

Fatigue index = (Amplitude ratio-AMP)/(Mean frequency-MF ratio), where AMP ratio = AMP during the 10-s period every minute following load application/AMP during the 10-s baseline period (measured immediately after load application); MF ratio = MF during the 10-s period every minutes following load application/MF during the 10-s baseline period. An example of the EMG recording and the evaluation of the fatigue index is reported in the Supplementary Materials.

This approach is based on methodologies described by Merletti and Lo Conte (1997) [43] and Schmid and Schieppati [13].

#### 2.2.6. Experimental Protocol

Participants were blinded to test conditions and instructed to fixate on an earth-fixed visual target for at least 1 min before testing. Tests of symmetric and asymmetric cycles in condition C, V, C + V were randomized and separated by at least 24 h. Each condition was tested before and after maximal or partial neck muscle fatigue. In an additional trial, the effect of light was assessed by performing three asymmetric cycles under illumination, followed by a fourth cycle in darkness in condition C + V.

#### 2.2.7. Data Acquisition and Analysis

The potentiometer and the chair signals were fed to the computer (sampling rate 50 Hz) for display and storage. The data were analyzed offline to evaluate the gain (amplitude of the perceptual responses/amplitude of the stimulus) for showing the efficacy of the proprioceptive and vestibular system in the self-motion perception and the tracking final position error (FPE) for showing the efficacy of these sensory signals in response to asymmetric rotation, during contrasting velocity stimulation. The responses were statistically analyzed using generalized mixed model analysis (GLM). The analysis included perceptual responses as the dependent variables; groups of tests before and under fatigue, the frequency of stimulation and interactions as the fixed effects of main interest; and a random effect for the repeated measures. This analysis allowed us to establish the statistical significance of the perceptual difference observed before and during neck muscle fatigue. The degree of convergence was good for all the statistical evaluations because the standard deviations and the standard error were low compared to the means that estimated average was very stable. Statistical post hoc analyses were conducted using Bonferroni correction for multiple comparisons. The eta-square (Ƞ^2^) was computed to assess effect size. The level of significance was set at *p* < 0.05 for both the GLM values and post hoc comparisons. The confidence intervals of the data are reported in the Supplementary Materials. Prior to GLM, the Shapiro‒Wilk test was used to assess normality, and Levene’s test was used to assess homogeneity of variance.

## 3. Results

### 3.1. Effect of Neck Muscle Fatigue on Self-Motion Perceptual Gain in Response to Head and Trunk Symmetric Sinusoidal Rotation

**Figure 2:**
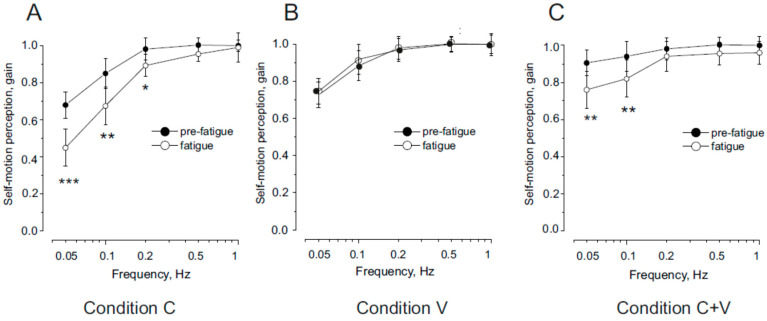
Self-motion perception gain (**A**) in response to symmetric sinusoidal trunk rotation with the head stationary (Condition C), (**B**) in response to whole-body rotation (Condition V), (**C**) in response to head rotation with the trunk stationary (Condition V + C). Data represent mean ± standard deviation (SD) of ten individuals before (filled symbols) and after (open symbols) neck muscle fatigue. Statistically significant differences between conditions are indicated: * *p* < 0.05; ** *p* < 0.01; *** *p* < 0.001.

In Figure 2A (Condition C), we report the effect of neck muscle fatigue on the gain of self-motion perception during trunk rotation with the head stationary (neck proprioceptive stimulation). Under baseline (non-fatigued) conditions, in line with previous studies [15,44,45], proprioceptive signals from the neck provided accurate self-motion perception, particularly at higher rotational frequencies. At lower frequencies, however, the perceptual response tended to decrease.

Following neck muscle fatigue, motion perception was significantly reduced at 0.05 and 0.1 Hz, whereas no significant changes were observed at higher frequencies.

Statistical analysis confirmed a significant reduction in gain following fatigue (F(1,36) = 29.15, *p* < 0.001, η^2^ = 0.88), with this effect being frequency-dependent. Specifically, gain decreased significantly at 0.05 Hz and 0.1 Hz (*p* < 0.001), and at 0.2 Hz (*p* < 0.05), but remained unchanged at 0.5–1 Hz (*p* = 0.36–0.55).

Figure 2B shows the results for **Condition V** (whole-body rotation, vestibular stimulation). In the non-fatigue condition, the gain profile condition following vestibular stimulation closely resembled that observed following the neck muscle stimulation, in line with previous studies [15,44,45]. Notably, neck muscle fatigue had no significant effect on self-motion perception: gain amplitudes before and after fatigue were statistically indistinguishable across all frequencies (F(1,36) = 0.52, *p* = 0.76, η^2^ = 0.79).

Figure 2C (Condition V + C) illustrates the gain of self-motion perception during head rotation with the trunk stationary—activating both vestibular and neck proprioceptive inputs. As previously shown [44,45], baseline perceptual gain remained high across both low and high frequencies.

However, after the fatigue procedure, a significant reduction in gain was observed (F(1,36) = 12.15, *p* < 0.01, η^2^ = 0.88), again with a frequency-dependent effect. Gain decreased significantly at lower frequencies (0.05–0.1 Hz, *p* < 0.001), but remained unchanged at higher frequencies (0.2–1 Hz, *p* = 0.27–0.45).

Effect of Neck Muscle Fatigue on Self-Motion Perceptual Final Position in Response to Asymmetric Sinusoidal Rotation of the Head and Trunk

**Figure 3:**
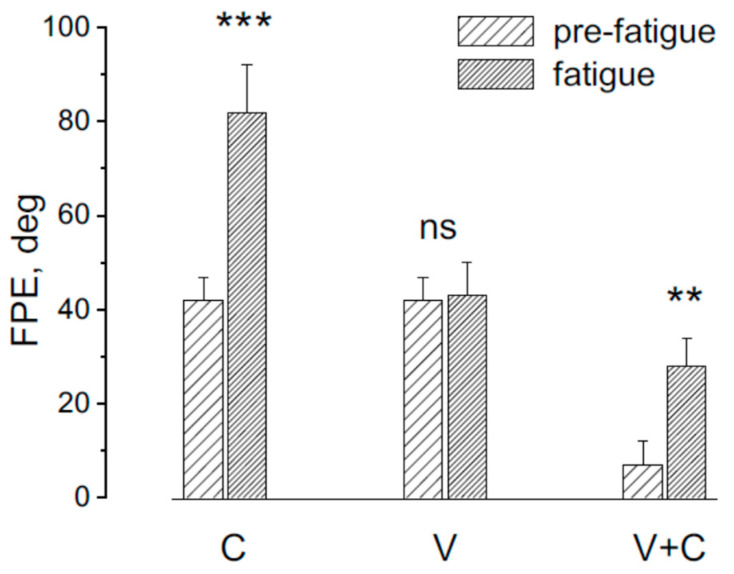
Final position error (FPE) in response to asymmetric rotation before and after the fatiguing procedure. In condition **C**, the trunk was rotated while the head remained stationary, primarily stimulating cervical proprioceptors. In condition **V**, the entire body was rotated, targeting vestibular input. In condition **V + C**, the head was rotated on a stationary trunk, stimulating both cervical and vestibular receptors. Note that in conditions **C** and **V + C**, fatigue significantly increased the FPE (*** *p* < 0.001, ** *p* < 0.01), whereas no significant change (ns) was observed in condition **V**.

The effect of asymmetric rotation on FPE was assessed before and after the fatiguing procedure across three experimental conditions: Condition (C); Condition (V); Condition + V (Figure 3).

Following the fatiguing procedure, a significant change in FPE was observed (F(2,18) = 13.15, *p* < 0.01, η^2^ = 0.78). Specifically, FPE increased significantly in response to trunk rotation (*p* < 0.001) and head rotation (*p* < 0.001), whereas no significant difference was found in response to whole-body rotation (*p* = 0.21)

### 3.2. Effect of Light on the Induction of FPE During Asymmetric Rotation

All ten participants underwent sequences of asymmetric head rotations while keeping their trunks stationary, under both light and dark conditions. The first three cycles served as conditioning trials, followed by a fourth test cycle conducted in darkness. This procedure was preceded by a neck muscle fatigue protocol.

**Figure 4:**
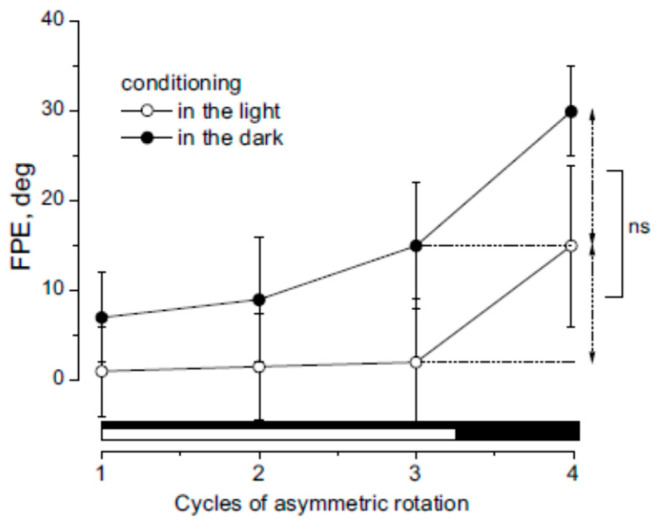
FPE was measured during the 4th asymmetric rotation cycle following conditioning with three prior cycles performed either in darkness (filled symbols, black bars) or in light (open symbols, white bars). Data are presented as mean ± SD for 10 participants. The increase in FPE induced by the 4th cycle (indicated by extension lines) was similar between light and dark conditioning, with no significant difference (ns).

Under dark conditioning, positional error progressively increased across the cycles and the FPE at the end of rotation was 39 ± 9°. This substantial increase has been attributed to cumulative adaptation to velocity contrast during asymmetric rotation [15,38], and, as previously reported [15,38], reflects a gradual reduction in sensitivity to slow motion (Figure 4).

In contrast, under light conditioning, motion perception remained accurate throughout the first three cycles (Final error: FPE = 0–2°). Nevertheless, in the fourth (dark) cycle, the FPE still increased to 15 ± 9°, despite the absence of perceptual error in the preceding cycles. Statistical analysis revealed a significant difference in FPE across cycles between the light- and dark-conditioning groups (F(1,27) = 19.11, *p* < 0.01, η^2^ = 0.82). However, the magnitude of perceptual error increase in the fourth cycle (ΔFPE: Cycle 4 FPE– Cycle 3 FPE) did not significantly differ between the two conditions (F(19) = 1.01, *p* = 0.49, η^2^ = 0.82). These findings suggest that adaptation occurred even during light conditioning, despite the absence of explicit perceptual errors in those cycles.

### 3.3. Effect of Different Fatigue Levels on FPE Induction During Asymmetric Rotation

The relationship between the degree of neck muscle fatigue and the magnitude of the final position error (FPE) was assessed in separate sessions conducted after only 1, 3 or 6 min of the fatiguing protocol. FPE, induced by asymmetric head rotation with the trunk held stationary, was examined in ten participants across the different experimental sessions.

**Figure 5:**
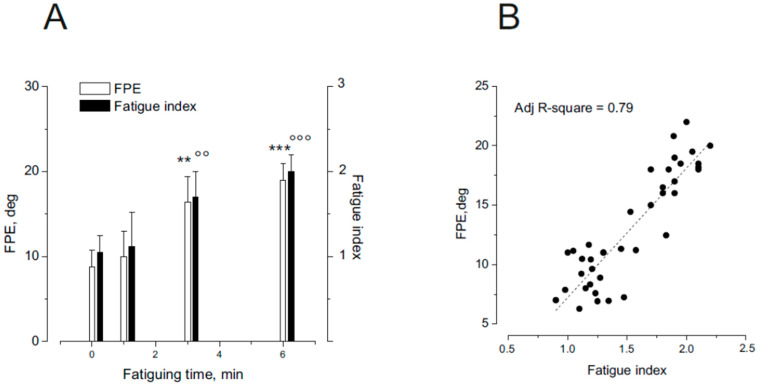
(**A**) Effect of different duration of neck muscle fatigue on FPE (1, 3, 6 min). The effect is compared with fatigue index from the EMG of the dorsal neck muscles. The FPE after fatiguing procedure was compared with the pre-fatigue from 10 individuals. Note that the FPE was higher than the normal after a fatiguing procedure lasting 3 min (*p* < 0.01 **, °°) and 6 min (*p* < 0.001 ***, °°°). (**B**) linear correlation between the fatigue index and the FPE amplitude.

As shown in Figure 5A, FPE increased significantly with the duration of fatigue (F(3,36) = 19.32, *p* < 0.001, η^2^ = 0.88). Specifically, after 1 min of fatigue, no significant changes were observed in either FPE or the fatigue index (in Supplementary Materials: recording of the dorsal neck muscle EMG during neck muscle fatigue and the evaluation of fatigue index). However, following 3 and 6 min of the fatiguing procedure, both FPE and the fatigue index were significantly elevated. In Figure 5B, the linear correlation between the fatigue index and the FPE is shown.

## 4. Discussion

### 4.1. Perceptual Impairment by Neck Muscle Fatigue During Head and/or Trunk Rotation

This study demonstrates that neck muscle fatigue impairs self-motion perception during slow sinusoidal head rotations, when the head moves on a stationary trunk. This impairment leads to a mis-localization of the self in relation to external space, especially after sequences involving alternating fast and slow head movements. The observed misperception is likely due to reduced proprioceptive sensitivity to low-velocity motion [15], which in turn diminishes perceptual gain during slow rotations. Consequently, motion perception becomes biased in favor of higher-velocity stimuli, resulting in a positional error at the conclusion of rotation. Although all participants exhibited fatigue-induced perceptual impairments, the magnitude of the deficit varied among individuals. However, due to the small sample size, it was not possible to establish any robust relationships between the extent of impairment and individual characteristics such as age, physical activity, or lifestyle factors.

Previous studies [15,16] reported perceptual errors only during trunk rotation with the head stationary, indicating that those distortions were driven purely by proprioceptive dysfunction. In contrast, our results show that self-motion perception is also compromised during head-on-trunk rotations, which engage both cervical proprioception and vestibular input. This finding suggests that the presence of vestibular input, which remains functionally intact during fatigue, is not sufficient to compensate for proprioceptive degradation. These results emphasize the critical contribution of cervical proprioceptive input to low-velocity motion perception, and show that vestibular signals, although accurate, are less effective in compensating for deficits at low dynamics.

Given that head movements occur far more frequently than trunk rotations in daily activities, these findings underscore the importance of neck proprioception in sensorimotor integration. Our findings also provide further indirect evidence for the deleterious impact of muscle fatigue on proprioceptive signaling, a mechanism previously proposed in animal studies [17,18] and directly demonstrated in humans [19,20,21,22,23,24,25,26,27,28,29,30,31,32,33,34,35,36].

### 4.2. Persistence of Perceptual Impairment Under Lighted Conditions

A second major finding of this study is that the perceptual distortions induced by neck muscle fatigue persist—even under lighted conditions. Although visual input is known to suppress perceptual errors during asymmetric stimulation, we observed that head-on-trunk rotations initially performed under lighted conditions, followed by testing in darkness, resulted in enhanced mis-localization. This occurred despite the absence of perceptual errors during the lighted conditioning phase.

These findings suggest that the central nervous system retains the effects of asymmetric stimulation even when perceptual errors are not overtly expressed due to the presence of visual feedback. Similar retention effects have been previously reported during vestibular-only asymmetric stimulation [46]. Importantly, this supports the notion that a dissociation exists between perceptual experience and underlying neural processes. Visual input appears to mask—but not eliminate—errors induced by proprioceptive or vestibular dysfunction. Thus, perceptual distortions arising from proprioceptive misperception are not only generated but also maintained during asymmetric motion, even when performed under visual guidance. When visual input is removed, the cumulative effects of earlier distortion become unmasked, leading to amplified errors in spatial localization.

### 4.3. Fatigue-Induced Errors at Moderate Levels of Muscle Fatigue

A third key finding is that perceptual distortions can be induced even by moderate levels of neck muscle fatigue. Previous studies have generally reported such misperceptions only after exhaustive or high-intensity fatiguing protocols. In contrast, our data show that even short durations of fatigue (e.g., 3 min) can produce statistically significant impairments in self-motion perception.

Moreover, we found that the magnitude of the perceptual error was proportional to the degree of fatigue, as quantified by EMG-based fatigue indices. These findings highlight the possibility that even everyday levels of muscle fatigue—such as those resulting from prolonged static postures or low-intensity repetitive movements—may be sufficient to disrupt motion perception.

## 5. Conclusions

In summary, this study provides compelling evidence that neck muscle fatigue significantly disrupts both self-motion and position perception, especially during low-velocity movements. These impairments persist even when other sensory systems such as vision and vestibular input remain functional, suggesting that proprioceptive degradation alone is sufficient to compromise spatial orientation.

Such perceptual distortions have clear implications for postural stability, balance control, and motor performance, increasing susceptibility to disorientation and movement errors.

One limitation of this study is the relatively small sample size. Although statistical analyses yielded results with high power, it remains possible that the selected sample—despite including participants with varied age, sex, and lifestyle—did not capture the full extent of inter-individual variability. Future studies with larger and more diverse populations are needed to determine whether the magnitude of perceptual impairment correlates with specific individual traits. Nonetheless, the present findings offer robust evidence that neck muscle fatigue can produce substantial sensorimotor disruption.

Given that neck fatigue is common in daily life—arising from factors such as poor posture, prolonged screen use, extended driving, non-ergonomic work conditions, and athletic activity—these results have broad relevance. At-risk populations include individuals who maintain prolonged neck extension (e.g., rock climbers), those wearing head-borne loads (e.g., helicopter pilots with heavy helmets), and athletes engaged in asymmetric head movements. Once a critical fatigue threshold is reached, these individuals may experience reduced spatial awareness, loss of balance, and impaired motor coordination—with potential consequences for safety and performance.

## Data Availability

Details regarding where data supporting reported results can be found in the lab of Department of Medicine and Surgery, Human Physiology Section, Università Degli Studi di Perugia, Piazzale Gambuli 1, 06100 Perugia, Italy.

## Supplementary Materials

The following supporting information can be downloaded at: https://www.mdpi.com/article/10.3390/audiolres15050128/s1. EMG form an individual. Above—Electromyographic recording from the neck extensor muscles during fatigue procedure (left side) and rectified EMG (right side). Below—median of the discharge frequency (left side) and the fatigue index (right side) during the fatiguing procedure. Statistical values of the data in pre and post fatigue reported in the Figure 2, Figure 3 and Figure 4: mean and confidence interval at different frequency of rotation for sinusoidal (Figure 2) and asymmetric (Figure 3) stimulation. From Figure 4 the data (mean and confidence interval) in dark and light conditioning.
