# Supplementary material for: Self-Motion Misperception Induced by Neck Muscle Fatigue
Source: Audiol Res. 2025 Oct 2;15(5):128. doi: 10.3390/audiolres15050128 (PMC12561854; doi:10.3390/audiolres15050128)
Supplement: Supplementary file 1 [file audiolres-15-00128-s001.zip › tabella statistica.pdf]

Sinusoidal rotation

Fig 2 A

Pre fatigue

Hz	means	and confidence interval	
0,05	0,67	0,61	0,73
0,1	0,85	0,79	0,91
0,2	0,98	0,93	1,03
0,5	1,01	0,91	1,11
1	1,02	0,92	1,12

Post fatigue

0,05	0,45	0,41	0,49
0,1	0,67	0,62	0,72
0,2	0,89	0,85	0,93
0,5	0,95	0,92	0,98
1	0,99	0,92	1,06

Fig 2 B

Pre fatigue

0,05	0,75	0,71	0,79
0,1	0,89	0,85	0,93
0,2	0,98	0,92	1,04
0,5	1,00	0,95	1,05
1	1,00	0,94	1,06

Post fatigue

0,05	0,74	0,70	0,78
0,1	0,91	0,86	0,96
0,2	0,97	0,92	1,02
0,5	1,00	0,95	1,05
1	1,00	0,96	1,04

Fig 2C

Pre fatigue

0,05 0,90 0,86 0,94

0,1 0,94 0,90 0,98

0,2 0,98 0,92 1,04

0,5 1 0,95 1,05

1 1 0,94 1,06

Post fatigue

0,05 0,76 0,72 0,80

0,1 0,82 0,78 0,86

0,2 0,94 0,90 0,98

0,5 0,95 0,90 1,00

1 0,96 0,96 1,02

Fig 3 Asymmetric rotation

C Pre fatigue 42 35 49

Post fatigue 82 70 94

V Pre fatigue 42 36 48

Post fatigue 43 35-51

VC Prefatigue 7 1 13

Postfatigue 28 20 36

Fig 4 Dark and light conditioning /asymmetric rotation)

Dark conditioning last cycle 15 10-20

Light conditioning last cycle 14 9 -19
