# Supplementary figures and images for: Self-Motion Misperception Induced by Neck Muscle Fatigue
Source: Audiol Res. 2025 Oct 2;15(5):128. doi: 10.3390/audiolres15050128 (PMC12561854; doi:10.3390/audiolres15050128)

## Record of dorsal neck muscle EMG during fatiguing procedure

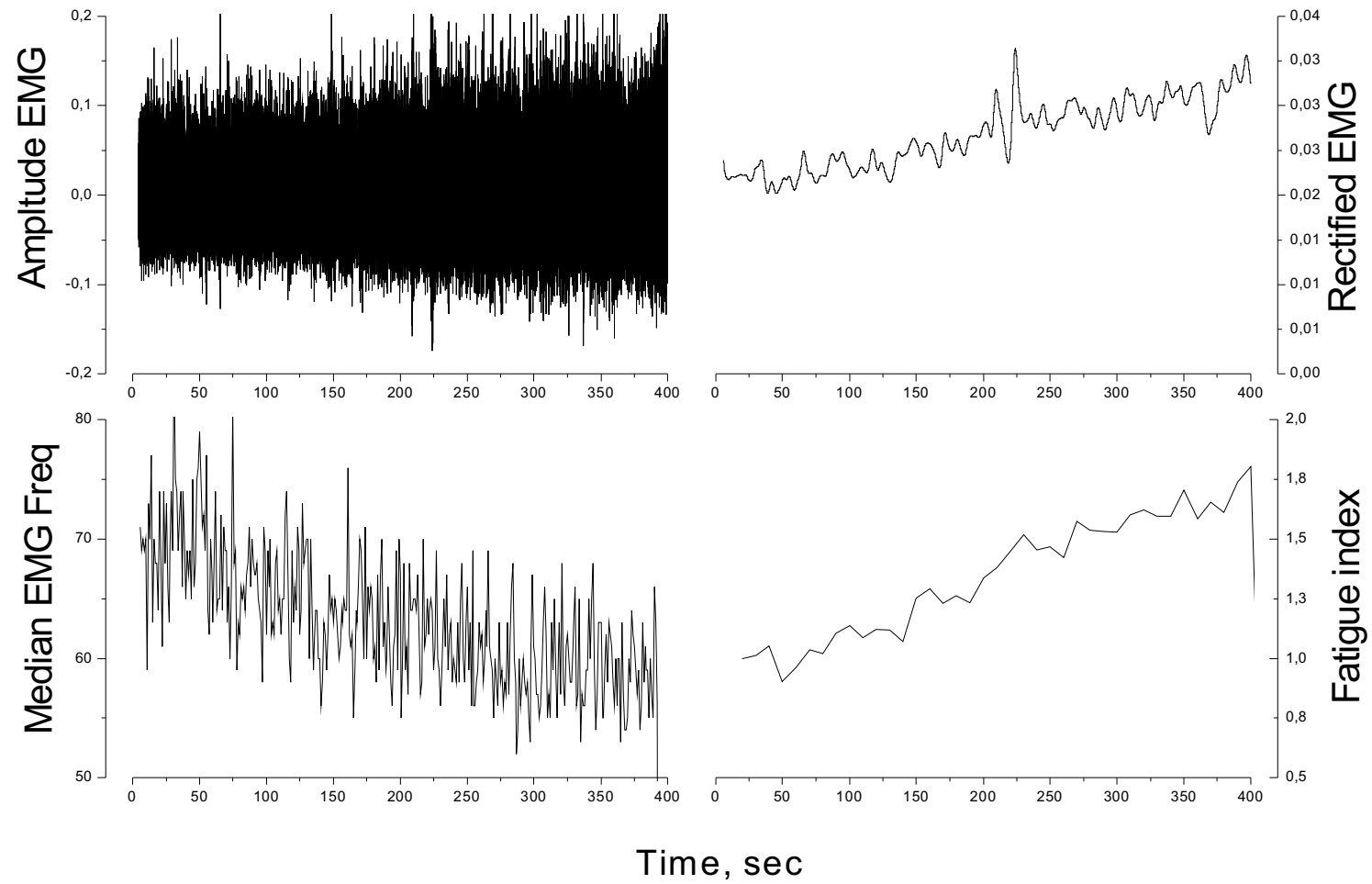

Supplement: Supplementary file 1 [file audiolres-15-00128-s001.zip › EMG.pdf]
